# Interaction of PLS and PIN and hormonal crosstalk in *Arabidopsis* root development

**DOI:** 10.3389/fpls.2013.00075

**Published:** 2013-04-05

**Authors:** Junli Liu, Saher Mehdi, Jennifer Topping, Jirí Friml, Keith Lindsey

**Affiliations:** ^1^The Integrative Cell Biology Laboratory and The Biophysical Sciences Institute, School of Biological and Biomedical Sciences, Durham University, Durham, UK; ^2^VIB Department of Plant Systems Biology and Department Plant Biotechnology and Bioinformatics, University of Gent, Gent, Belgium

**Keywords:** hormonal crosstalk, root development, auxin flux, PIN proteins, PLS protein, signaling network

## Abstract

Understanding how hormones and genes interact to coordinate plant growth is a major challenge in developmental biology. The activities of auxin, ethylene, and cytokinin depend on cellular context and exhibit either synergistic or antagonistic interactions. Here we use experimentation and network construction to elucidate the role of the interaction of the POLARIS peptide (PLS) and the auxin efflux carrier PIN proteins in the crosstalk of three hormones (auxin, ethylene, and cytokinin) in *Arabidopsis* root development. In ethylene hypersignaling mutants such as *polaris* (*pls*), we show experimentally that expression of both PIN1 and PIN2 significantly increases. This relationship is analyzed in the context of the crosstalk between auxin, ethylene, and cytokinin: in *pls*, endogenous auxin, ethylene and cytokinin concentration decreases, approximately remains unchanged and increases, respectively. Experimental data are integrated into a hormonal crosstalk network through combination with information in literature. Network construction reveals that the regulation of both PIN1 and PIN2 is predominantly via ethylene signaling. In addition, it is deduced that the relationship between cytokinin and PIN1 and PIN2 levels implies a regulatory role of cytokinin in addition to its regulation to auxin, ethylene, and PLS levels. We discuss how the network of hormones and genes coordinates plant growth by simultaneously regulating the activities of auxin, ethylene, and cytokinin signaling pathways.

## INTRODUCTION

Hormone signaling systems coordinate plant growth and development through a range of complex interactions. The activities of auxin, ethylene, and cytokinin depend on cellular context and exhibit either synergistic or antagonistic interactions. Additionally, auxin is directionally transported through plant tissues, providing positional and vectorial information during development (Vanneste and Friml, 2009). Hormones and the associated regulatory and target genes form a network in which relevant genes regulate hormone activities and hormones regulate gene expression (Chandler, 2009; Depuydt and Hardtke, 2011; Vanstraelen and Benkov, 2012). In addition, hormones also interact with other signals such as glucose to control root growth (Kushwah et al., 2011). An important question for understanding these complex interactions is: what are the mechanisms that regulate the fluxes of plant hormones and levels of the proteins encoded by the regulatory and target genes?

Patterning in *Arabidopsis* root development is coordinated via a localized auxin concentration maximum in the root tip (Sabatini et al., 1999), requiring the regulated expression of specific genes. This auxin gradient has been hypothesized to be sink-driven (Friml et al., 2002) and computational modeling suggests that auxin efflux carrier activity may be sufficient to generate the gradient in the absence of auxin biosynthesis in the root (Grieneisen et al., 2007; Wabnik et al., 2010). However, other experimental studies show that local auxin biosynthesis modulates gradient-directed planar polarity in *Arabidopsis*, and a local source of auxin biosynthesis contributes to auxin gradient homeostasis (Ikeda et al., 2009). Thus genetic studies show that auxin biosynthesis (Ikeda et al., 2009; Normanly, 2010; Zhao, 2010), the AUX1/LAX influx carriers (Swarup et al., 2005, 2008; Jones et al., 2008; Krupinski and Jonsson, 2010), and the PIN auxin efflux carriers (Petrásek et al., 2006; Grieneisen et al., 2007; Krupinski and Jonsson, 2010; Mironova et al., 2010) all play important roles in the formation of auxin gradients. Since auxin concentration is regulated by these and diverse interacting hormones, it cannot change independently of these various components in space and time.

Therefore, a quantitative understanding of the effects of any perturbation experiment on auxin gradients and root development (e.g., genetic perturbations or exogenously applied hormones) must be analyzed in the context of hormonal interactions. For example, ethylene promotes auxin flux in the root, in a process dependent on the POLARIS (PLS) peptide (Ruzicka et al., 2007; Swarup et al., 2007; Liu et al., 2010). Furthermore, PIN levels are positively regulated by ethylene and auxin in *Arabidopsis* roots (Ruzicka et al., 2007). Interestingly, cytokinin can negatively regulate PIN levels (Ruzicka et al., 2009), while repressing auxin biosynthesis and promoting ethylene responses (Nordstrom et al., 2004; Chandler, 2009; Liu et al., 2010). Cytokinin also has the capacity to modulate auxin transport, by transcriptional regulation of the *PIN* genes (Ruzicka et al., 2009).

We previously developed a hormonal interaction network for a single *Arabidopsis* cell by iteratively combining modeling with experimental analysis (Liu et al., 2010). We described how such a network regulates auxin concentration in the *Arabidopsis* root, by controling the relative contribution of auxin influx, biosynthesis and efflux; and by integrating auxin, ethylene, and cytokinin signaling. Here we integrate PIN-mediated auxin flux with interacting hormone signaling modules. Specifically, we build on the hormonal crosstalk model (Liu et al., 2010) and construct a network to describe interaction of PLS and PIN proteins and hormonal crosstalk in *Arabidopsis* root development, using experimental data in the literature and our measurements.

## RESULTS

### RELATIONSHIP BETWEEN AUXIN, ETHYLENE, CYTOKININ, AND PLS

Our previous experimental measurements have shown the following response of auxin, ethylene, cytokinin to *PLS* expression. In the *polaris *(*pls*) mutant, auxin concentration decreases, cytokinin concentration increases and ethylene concentration remains approximately unchanged (Casson et al., 2002; Chilley et al., 2006; Liu et al., 2010). In the *PLS *overexpressing transgenic* PLOSox*, auxin concentration increases, and ethylene concentration remains approximately unchanged. In the *pls etr1* double mutant, auxin concentration is approximately recovered to the same level as that in wild-type seedlings.

In addition, the exogenous application of indole acetic acid (IAA) to wild-type seedlings increases both endogenous auxin concentration and *PLS* expression, while exogenous application of cytokinin to wild-type seedlings decreases both endogenous auxin concentration and PLS expression. Moreover, when 1-aminocyclopropane-1-carboxylic acid (ACC) is exogenously applied to wild-type seedlings, auxin concentration increases, but *PLS* expression decreases. However, in *pls*, although endogenous auxin concentration is lower than that in wild-type, the exogenous application of ACC further decreases auxin concentration (Chilley et al., 2006; Liu et al., 2010).

Therefore, PLS has a role in the crosstalk between auxin, ethylene, and cytokinin. By iteratively combining modeling with experimental analysis (Liu et al., 2010), we developed a hormonal crosstalk network. We described how such a network regulates auxin concentration in the *Arabidopsis* root, by controling the relative contribution of auxin influx, biosynthesis and efflux; and by integrating auxin, ethylene, and cytokinin signaling.

### EXPERIMENTAL MEASUREMENTS OF THE RELATIONSHIP BETWEEN PINs AND PLS

**FIGURE 1:**
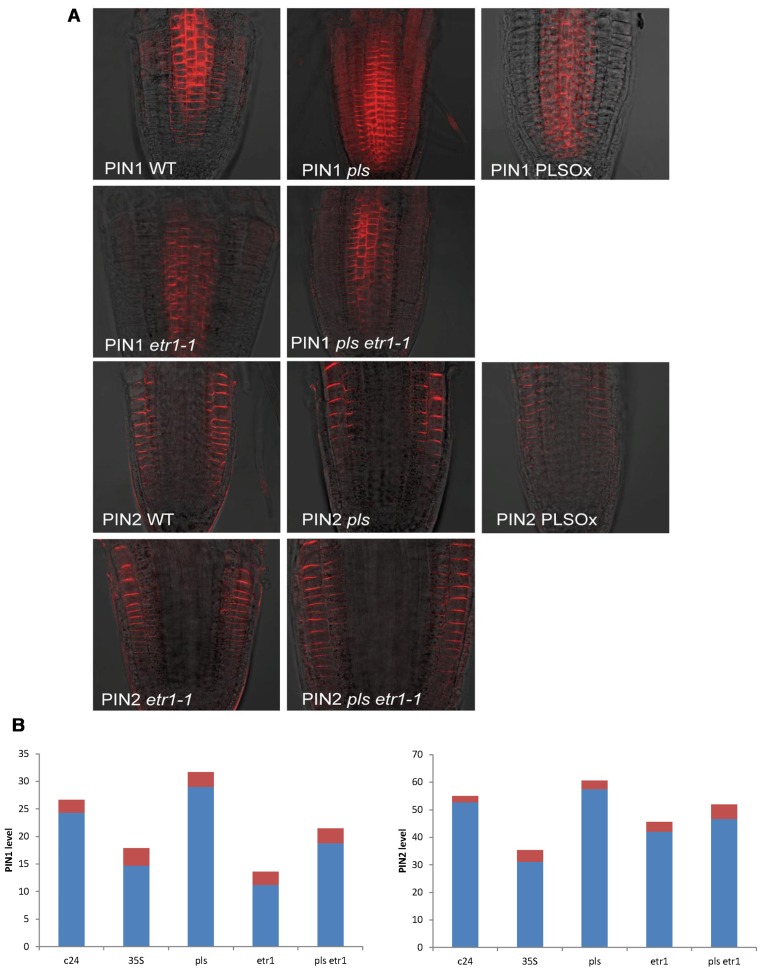
**(A) PIN1 and PIN2 immunolocalization in wild-type, *pls*, *PLSox*, *etr1*, and *etr1 pls* double mutants of *Arabidopsis*, showing differences in PIN protein levels**. **(B)** Quantification of PIN1 and PIN2 levels in wild-type, *pls*, *PLSox*, *etr1* and *etr1 pls* double mutants in *Arabidopsis*. The red colored bars represent the standard errors of the mean (*n* = 10).

Here we experimentally determined PIN1 and PIN2 protein levels in the seedling root of wild-type, *pls* mutant, *PLSox*, *etr1*mutant, and *pls etr1* double mutant (**Figure 1**). Immunolocalization studies revealed that both PIN1 and PIN2protein levels increase in the *pls* mutant, and decrease in *PLSox*. In the ethylene-insensitive *etr1* mutant, PIN1 and PIN2 levels are lower than in wild-type. In addition, the double mutant *pls etr1* exhibits reduced PIN1 and PIN2 levels compared to *pls* and slightly lower PIN1 and PIN2 levels compared to wild-type.

These experimental data show that PLS and PIN1/PIN2 form an interaction network, which regulates hormonal crosstalk between auxin, ethylene, and cytokinin. Previously, we were able to model interactions between auxin, ethylene, and cytokinin (Liu et al., 2010). Here we describe an expanded network that integrates the interactions between these hormones and PIN auxin transporters, based on the newly identified relationship between PLS and PINs (**Figures 1A,B**) and previous experimental data on ethylene effects on auxin transport (Ruzicka et al., 2007; Swarup et al., 2007) and PLS effects on ethylene responses (Chilley et al., 2006). All the analysis in this work is applicable to both PIN1 and PIN2, and we use the term PIN generically. We do not consider other forms of PINs, as our experiments and modeling focus on the auxin fluxes through the plasma membrane in this work.

### NETWORK FOR INTERACTION OF PIN AND PLS AND HORMONAL CROSSTALK

**FIGURE 2:**
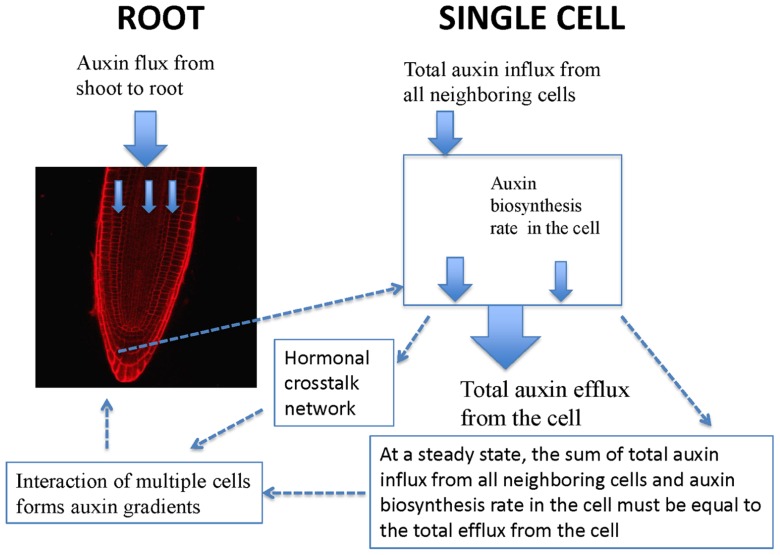
**Schematic description of the relationship between auxin spatial gradients and interaction of PIN and PLS as well as hormonal crosstalk at a cellular level**. In a single cell, PIN and PLS interact and hormonal crosstalk occurs. At a steady state, the sum of total auxin influx from all neighboring cells and auxin biosynthesis rate in the cell must be equal to the total efflux from the cell. Moreover, when multiple cells interact, auxin gradients emerge.

Experimentally measured data (**Figure 1A**) are applicable for tissues rather than for a single cell. PIN1 and PIN2 levels in **Figure 1B** are the overall levels of the whole tissues. However, the interaction of PIN and PLS is at the cellular level. In order to use experimental data to analyze the interaction of PIN and PLS at a cellular level, the data for tissues have to be linked to the interactions in each cell (**Figure 2**). To do this, the following assumptions are made. First, all measured data are at steady states. Second, all fluxes or concentrations are relative to the respective counterparts in wild-type. If the auxin flux from shoot to root is increased or reduced, the influx in a single cell is considered to be correspondingly increased or reduced. This is because, at a steady state, the sum of total auxin influx from all neighboring cells and auxin biosynthesis rate in the cell must be equal to the total auxin efflux from the cell (**Figure 2**). Therefore, for all connecting cells in a tissue, the auxin flux from shoot to root affect the influx of all cells. A third assumption is that, when the level of PIN is compared, we assume the location of PIN does not change. For example, in the *pls* mutant, both PIN1 and PIN2protein levels increase (**Figure 1**). We consider this occurs at the original location of PIN1 and PIN2.

At a cellular level, PIN and PLS interact and a hormonal crosstalk network forms (**Figure 2**). Auxin fluxes and biosynthesis rates are regulated by all components in the network. At a tissue level, multiple cells interact and auxin gradients emerge (**Figure 2**). The current analysis concentrates on the study of the regulatory network for hormonal crosstalk: namely how PIN and PLS interacts at a cellular level and how hormonal crosstalk occurs. The spatial distribution of auxin in the root is due to spatial setting of PIN in multiple interacting cells, as modeled by Grieneisen et al. (2007).

In order to analyze the interaction of PIN and PLS and crosstalk with other hormonal signaling systems, we integrate the newly identified relationship between PLS and PIN (**Figure 1**) with the experimental data in the literature. When these data are incorporated into the network (Liu et al., 2010), two regulatory relationships emerge: first, that ethylene signaling promotes PIN levels; and second, that a decrease in PIN levels occurs following exogenous application of cytokinin (Ruzicka et al., 2009). Network construction for the interactions between hormonal pathways and PIN protein levels is described as follows.

**FIGURE 3:**
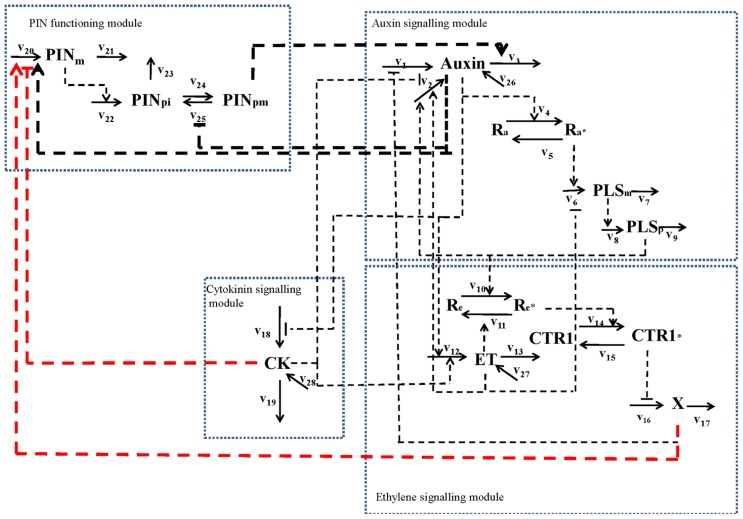
**Network for the interaction of PIN and PLS and hormonal crosstalk in the situation in which cytokinin decreases endogenous auxin concentration** (Eklof et al., 1997; Nordstrom et al., 2004). The network includes four modules: an auxin signaling module; an ethylene signaling module; a cytokinin signaling module; and a PIN function module. In a previous study (Liu et al., 2010), the first three modules were described in detail. In this work, we integrate the PIN function module with the three hormone signaling modules. The three thick black dashed lines represent the regulatory relationships directly supported by experimental evidence: auxin positively regulates PIN (e.g., PIN1) levels and transcriptional effects associated with auxin regulation were identified; PIN internalization is inhibited by auxin; auxin efflux carrier activity (PIN1 and PIN2) positively regulates auxin transport. The two thick red dashed lines represent the regulatory relationships derived by the combined analysis of experimental evidence and the hormonal crosstalk network described previously (Liu et al., 2010). The reaction rates are: v1: total auxin influx from all neighboring (**Figure 2** and text for details); v2: auxin biosynthesis rate in the cell; v3: total auxin efflux from the cell; v4: rate for conversion of the inactive form of the auxin receptor to its active form; v5: rate for conversion of the active form of the auxin receptor to its inactive form; v6: transcription rate of the *POLARIS *(*PLS*) gene; v7: decay rate of *PLS* mRNA; v8: translation rate of the PLS protein; v9: decay rate of PLS protein; v10: rate for conversion of the inactive form of the ethylene receptor to its active form by PLS protein (PLSp); v11: rate for conversion of the active form of ethylene receptor to its inactive form; v12: ethylene biosynthesis rate; v13: rate for removal of ethylene; v14: rate for conversion of the inactive form of the CONSTITUTIVE TRIPLE RESPONSE 1 (CTR1) protein to its active form; v15: rate for conversion of the active form of CTR1 protein to its inactive form; v16: rate for activation of the ethylene signaling response; v17: rate for removal of the unknown ethylene signaling component, X; v18: rate for cytokinin biosynthesis; v19: rate for removal of cytokinin; v20: transcription rate of the *PIN* gene; v21: rare for the decay of *PIN *mRNA; v22: translation rate of PIN protein; v23: rate for decay of PIN protein in cytosol; v24: rate for transport of PIN protein from cytosol to plasma membrane; v25: rate for internalization of PIN protein. When exogenous hormones are applied: v26: rate for uptake of IAA when exogenous IAA is applied; v27: rate for uptake of ACC when exogenous ACC is applied; v28: rate for uptake of cytokinin when exogenous cytokinin is applied.

First, an increase in PIN level (**Figure 1**) and the observed simultaneous decrease in auxin concentration in the *pls *mutant (Chilley et al., 2006; Liu et al., 2010) imply that ethylene signaling also regulates PIN levels. This regulatory relationship is derived as follows. Experimentally, it has been shown that exogenous application of IAA and ACC can each increase PIN transcription and protein levels at the plasma membrane (Paciorek et al., 2005; Vanneste and Friml, 2009)*.* However, exogenous application of cytokinin reduces PIN levels (Ruzicka et al., 2009). Moreover, exogenous application of IAA or ACC increases endogenous auxin concentration, as shown by experimental data (Stepanova et al., 2007; Ruzicka et al., 2009) and as analyzed by modeling analysis (Liu et al., 2010). Furthermore, exogenous application of cytokinin decreases endogenous auxin concentration (Eklof et al., 1997; Nordstrom et al., 2004). In contrast, a recent report shows that exogenous application of cytokinin promotes auxin biosynthesis in young, developing tissues (Jones et al., 2010). We construct networks for both effects of cytokinin, based on biological knowledge and our own experimental observations. **Figure 3** shows the case that cytokinin decreases endogenous auxin concentration. For the case in which cytokinin promotes auxin biosynthesis, the network is exactly the same as in **Figure 3** except for the positive regulation of cytokinin to auxin biosynthesis.

As exogenous application of ACC increases both PIN levels and endogenous auxin concentration (Ruzicka et al., 2007), and as exogenous application of cytokinin decreases both PIN levels (Ruzicka et al., 2009) and endogenous auxin concentration (Nordstrom et al., 2004), one possibility is that exogenous ACC and cytokinin exert their effects on PINs by affecting endogenous auxin concentration. However, in *pls*, an increase in PIN levels (**Figures 1A,B**) corresponds to a decrease in auxin concentration (Figure 4C in Chilley et al., 2006). This indicates that auxin is not the only regulator of PIN levels, as otherwise PIN levels should decrease in *pls*. Therefore, ethylene signaling also regulates auxin efflux and this is realized by its regulation of PIN levels (Liu et al., 2010). The decrease in auxin concentration (Figure 4C in Chilley et al., 2006) and the increase in ethylene signaling in *pls* have opposite effects on PIN levels: the reduced auxin concentration that decreases PLS expression in turn reduces PIN levels, while the increase in ethylene signaling increases PIN levels (Chilley et al., 2006; Liu et al., 2010). The net effect is an increase in PIN levels. Therefore, when *PLS* expression changes, effects of ethylene signaling on PINs are more dominant than the effects of auxin. Experimental work (Chilley et al., 2006) and modeling (Liu et al., 2010) show that, in *pls*, endogenous ethylene concentration (evolution) is the same as in wild-type. Therefore, PLS regulates ethylene signaling rather than its synthesis, possibly due to interaction between PLS protein and ETHYLENE RESISTANT 1 (ETR1) (Chilley et al., 2006; Liu et al., 2010). In addition, the relationship between PIN levels and *pls*, *etr1*, and *pls etr1* double mutants supports the (at least genetic, if not physical) interaction between PLS and ETR1: In *pls* and* etr1*, PIN protein levels increase and decrease, respectively. Moreover, the double mutant *pls etr1* exhibits reduced PIN levels compared to *pls*, but increased PIN levels compared to *etr1* (**Figure 1**).

Therefore, the positive regulation of *PIN* expression by ethylene signaling is included in the network (**Figure 3**). The inclusion of this regulation is consistent with the experimental observations following exogenous application of IAA and ACC. When IAA is exogenously applied, both *PLS* expression and ethylene responses increase (Casson et al., 2002; Stepanova et al., 2007; Liu et al., 2010). Increasing *PLS* expression leads to decreased ethylene signaling, while increasing ethylene concentration increases ethylene responses. Therefore, application of exogenous IAA has antagonistic effects on ethylene signaling that regulates PIN levels. In addition, the increase of auxin concentration due to exogenous IAA application increases PIN levels. The overall effects of exogenous application of IAA lead to an increase in PIN levels. When ACC is exogenously applied, both endogenous ethylene and auxin concentrations increase, and *PLS* expression levels decrease (Chilley et al., 2006; Liu et al., 2010). Increase in ethylene concentration and decrease in PLS expression synergistically enhance ethylene responses. Therefore, when ACC is exogenously applied, auxin, ethylene and PLS all synergistically enhance PIN levels. Therefore, exogenous application of ACC leads to an increase in PIN levels.

The relationship between cytokinin and PIN levels implies an additional regulatory role of cytokinin in addition to its regulation to auxin, ethylene and PLS levels. This regulatory relationship is derived as follows. When cytokinin is exogenously applied, both endogenous cytokinin and ethylene concentrations increase, but *PLS* expression decreases (Liu et al., 2010). However, there are two opposite experimental observations for cytokinin effects: endogenous auxin either decreases (Eklof et al., 1997; Nordstrom et al., 2004) or increases (Jones et al., 2010).

Both decreased PLS protein and increased ethylene concentration synergistically enhance ethylene signaling (Casson et al., 2002; Liu et al., 2010). Following our analysis above, this increases PIN levels. When cytokinin positively regulates auxin biosynthesis (Jones et al., 2010), exogenous application of cytokinin increases endogenous auxin concentration and this positively regulates PIN levels. Therefore, when cytokinin is exogenously applied, changes in auxin, ethylene, and *PLS* expression all lead to the increase in PIN levels. However, it has been shown experimentally that exogenous application of cytokinin results in a reduction of PIN levels (Ruzicka et al., 2009). This implies that cytokinin has an additional role in regulating PIN levels, in addition to its regulation of auxin, ethylene, and PLS levels.

When cytokinin negatively regulates auxin biosynthesis, exogenous application of cytokinin decreases endogenous auxin concentration (Eklof et al., 1997; Nordstrom et al., 2004). The decrease in auxin concentration reduces PIN levels. However, the decrease in *PLS* expression and the increase in ethylene simultaneously enhance PIN levels. In the *pls* mutant, auxin concentration is low and ethylene concentration remains approximately unchanged (Liu et al., 2010). As analyzed above, due to the strong interaction between PLS protein and ETR1, PIN levels increase (**Figures 1A,B**) even though the auxin concentration has been reduced to a large extent (Petrásek et al., 2006; Figure 4C in Chilley et al., 2006). Based on experimental data (Chilley et al., 2006), we estimate that in *pls *roots, auxin concentration is 0.14 μM, compared with 0.23 μM in wild-type (Liu et al., 2010). Following exogenous application of cytokinin, an additional factor, i.e., an increase in ethylene concentration, also enhances ethylene signaling responses. Therefore, PIN levels should increase. However, experimental work shows that exogenous application of cytokinin results in the reduction of PIN levels (Ruzicka et al., 2009). Therefore, an explanation of the experimental results requires an additional regulatory role for cytokinin in controling PIN levels, and this is included in **Figure 3**.

In addition, PIN endocytic internalization is inhibited by auxin (Paciorek et al., 2005). Therefore, we have included in the network the inhibition by auxin of the cycling between PINpm (PIN at plasma membrane) to PINpi (PIN in cytosol). Therefore, by integrating our experimental data (**Figure 1**) with the experimental data in the literature, a hormonal crosstalk network of auxin, cytokinin and ethylene is revealed (**Figure 3**).

### HORMONAL CROSSTALK NETWORK AND ROOT GROWTH

As described in **Figures 2** and **3**, the concentrations of all three hormones (auxin, ethylene, and cytokinin) in root growth are mutually regulated by a hormonal crosstalk network. Therefore, they cannot change independently. Any genes that affect either the transport or biosynthesis of one of the three hormones have roles in the concentrations of all three hormones, as we demonstrated for the interaction of PIN and PLS. Auxin distribution is a versatile mechanism mediating a broad range of developmental responses (Petrásek and Friml, 2009). Both ethylene and cytokinin have roles in cell division (Ortega-Martínez et al., 2007; Dello Ioio et al., 2007). Therefore, an improved understanding of the roles of hormones and genes in root growth requires the analysis of hormonal crosstalk in space and time. For example, in *pls*, root elongation rate is slower than in wild-type (Casson et al., 2002). Due to the action of the hormonal crosstalk network (**Figure 3**) and as evidenced by experimental measurements, auxin concentration decreases, cytokinin concentration increases and ethylene concentration remains approximately unchanged (Casson et al., 2002; Chilley et al., 2006; Liu et al., 2010). As auxin concentration regulates elongation (Liu et al., 2010) and cytokinin concentration regulates the rates of cell division (Dello Ioio et al., 2007), the reduction of root elongation rate in *pls* is due to the changes in both auxin and cytokinin concentrations, as ethylene concentration remains approximately unchanged in *pls*.

## DISCUSSION

Transport-mediated, differential auxin distribution is a versatile mechanism mediating a broad range of developmental responses (Petrásek and Friml, 2009). The PIN-based auxin transport network can integrate various endogenous and environmental signals that modulate polarity or subcellular trafficking of PIN proteins, which are considered to be major regulatory mechanisms for PIN activity (Kleine-Vehn and Friml, 2008; Grunewald and Friml, 2010).

Nonetheless, experimental analyses have shown also that PIN levels in *Arabidopsis* vary in response to a range of hormones. Auxin positively regulates levels of several PIN proteins in different developmental contexts (Blilou et al., 2005; Laskowski et al., 2006; Chapman and Estelle, 2009; Vanneste and Friml, 2009) by a signaling pathway regulating transcription (Woodward and Bartel, 2005). Ethylene also upregulates PINs (e.g., PIN2) to remove auxin from the more distal region of the root tip (Ruzicka et al., 2007). Moreover, cytokinin negatively regulates PIN levels (Ruzicka et al., 2009). It is also evident that ethylene activates the biosynthesis of auxin locally in the root tip (Stepanova et al., 2007; Swarup et al., 2007), and that both auxin and cytokinin can synergistically activate the biosynthesis of ethylene (Chilley et al., 2006; Stepanova et al., 2007).

However, ethylene can also be synthesized without exogenous auxin and cytokinin application, such as in its role in root hair production (Tanimoto et al., 1995). When PIN levels change following a change in the concentration/response of a given hormone, it does not necessarily mean that the given hormone predominantly regulates PIN levels. This is because changing the concentration/response of a given hormone may also change the concentrations/responses of other hormones. As shown in this work, PIN levels are simultaneously regulated by auxin, ethylene, and cytokinin via the action of PLS. Therefore, PINs and hormones form an entangled network, and any perturbation in the network will cause changes in other components. As a result, auxin concentration is regulated by these and diverse interacting hormones via a hormonal crosstalk network, as demonstrated in the **Figure 3**.

This work demonstrates that integration of experimental measurements with existing knowledge in the literature is able to reveal how PIN1, PIN2, and three hormones (auxin, ethylene, and cytokinin) form an entangled network via the action of PLS. Our methodology involves two major steps. First, the PIN levels are measured (**Figure 1A**) and quantified (**Figure 1B**). Quantification of images shows the trends of the PIN levels (**Figure 1B**). Second, integrating experimental trends into existing knowledge reveals the crosstalk of PIN1, PIN2, auxin, ethylene, and cytokinin via the action of PLS. As all components in **Figure 3** form an entangled network, changing one component leads to changes in the others. Therefore, we propose that, in order to reveal the key regulatory points in the network, novel modeling methodology should be developed to dissect the regulation of the hormonal crosstalk network in the future.

The *Arabidopsis* genome contains eight *PIN* genes (Grunewald and Friml, 2010; Peer et al., 2011). Different PINs may have different locations and they may play different roles in auxin biology (Grunewald and Friml, 2010; Peer et al., 2011). For example, PIN1 and PIN2 exhibit primarily polar localizations on the plasma membrane while PIN3, PIN4, and PIN7 exhibit both polar and apolar plasma membrane localizations (Peer et al., 2011). In addition, hormones may regulate PIN levels differentially. For example, cytokinin can negatively regulate levels of PIN1, PIN2 and PIN3, but it positively regulates PIN7 levels (Ruzicka et al., 2007). In the current paper we construct the interaction network of PIN1, PIN2, auxin, ethylene, and cytokinin via the action of PLS. Following the methodology developed in this work, the interaction networks between other PINs, hormones and other genes could be constructed by measuring data similar to those described in **Figures 1A,B**. Moreover, as described in **Figure 2**, populating the hormonal crosstalk network in a spatial setting should be able to further model how auxin gradients are dependent on hormonal crosstalk in root development.

In addition, other phytohormones such as gibberellin and brassinosteroids are also important signals in the regulation of root development (Depuydt and Hardtke, 2011; Garay-Arroyo et al., 2012; Vanstraelen and Benkov, 2012). Although the effects of gibberellin and brassinosteroids on root development have been subjected to mathematical modeling studies (Middleton et al., 2012; van Esse et al., 2012), the networks describing their crosstalk with other hormones have not been constructed. The principle developed in this work can be used to further integrate the hormonal crosstalk for other phytohormones and genes in the future.

## MATERIALS AND METHODS

### PLANT MATERIALS

Wild-type (Col-0, C24) ecotypes and the *pls* and *pls etr1* mutants of *Arabidopsis thaliana* have been described previously (Topping and Lindsey, 1997; Casson et al., 2002; Chilley et al., 2006). *pls* DR5::GFP seedlings were generated by crossing (Liu et al., 2010). For *in vitro* growth studies, seeds were stratified, surface-sterilized and plated on growth medium (half-strength Murashige and Skoog medium (Sigma, Poole, UK), 1% sucrose, and 2.5% phytagel (Sigma) at 22 ± 2°C as described (Casson et al., 2009). For silver application experiments, seeds were germinated aseptically on growth medium or growth medium containing 10 μM silver nitrate.

### MICROSCOPY AND IMAGE ANALYSIS

Confocal images (for GFP imaging) were taken with a Bio-Rad Radiance 2000 microscope (Bio-Rad, Hemel Hempstead, UK) after counterstaining tissues with 10 mg/ml propidium iodide as described (Casson et al., 2009).

For comparisons of PIN protein signal intensities, at least three independent experiments were carried out. For each experiment at least 10 roots were evaluated with five random regions selected for signal intensity quantification for each. All fluorescence signals were evaluated on a Zeiss LSM 5 Exciter or Leica TCS SP2 confocal laser scanning microscopes. The same microscope settings were used for each independent experiment, and pixel intensities were taken into account when the images between controls and samples were compared. The average fluorescence intensity was measured with ImageJ (National Institutes of Health, http://rsb.info.nih.gov/ij). Statistics were evaluated with Excel (Microsoft). Results were visualized as average intensities with error bars representing standard deviation of the mean.

## Conflict of Interest Statement

The authors declare that the research was conducted in the absence of any commercial or financial relationships that could be construed as a potential conflict of interest.
